# Novel photoinduced phase transitions in transition metal oxides and diluted magnetic semiconductors

**DOI:** 10.1186/1556-276X-7-582

**Published:** 2012-10-23

**Authors:** Takashi Mizokawa

**Affiliations:** 1 Department of Complexity Science and Engineering, University of Tokyo, 5-1-5 Kashiwanoha, Kashiwa, Chiba, 277-8561, Japan

**Keywords:** Transition metal oxides, Photoemission spectroscopy, Photoinduced phase transition

## Abstract

Some transition metal oxides have frustrated electronic states under multiphase competition due to strongly correlated *d* electrons with spin, charge, and orbital degrees of freedom and exhibit drastic responses to external stimuli such as optical excitation. Here, we present photoemission studies on Pr_0.55_(Ca_1 − *y*_Sr_*y*_)_0.45_MnO_3_ (*y* = 0.25), SrTiO_3_, and Ti_1 **−** *x*_Co_*x*_O_2_ (*x* = 0.05, 0.10) under laser illumination and discuss electronic structural changes induced by optical excitation in these strongly correlated oxides. We discuss the novel photoinduced phase transitions in these transition metal oxides and diluted magnetic semiconductors on the basis of polaronic pictures such as orbital, ferromagnetic, and ferroelectric polarons.

## Background

In metal oxides and diluted magnetic semiconductors containing transition metal ions, correlated transition metal *d* electrons with spin, charge, and orbital degrees of freedom provide surprisingly rich electric and magnetic properties. In particular, electron–electron and electron-lattice interactions for the *d* electrons produce frustrated electronic states under multiphase competition which exhibit drastic responses to external stimuli such as optical excitation. Various photoinduced phase transitions have been reported in transition metal oxides such as Pr_0.55_(Ca_1 − y_Sr_y_)_0.45_MnO_3_[1] and in diluted magnetic semiconductors including In_1 − *x*_Mn_*x*_As [2]. In Pr_0.55_(Ca_1 − *y*_Sr_*y*_)_0.45_MnO_3_, the optically excited electrons locally destroy the spin-charge-orbital ordering, followed by rapid expanding of the disordered state for a persistent phase transition. On the other hand, in the diluted magnetic semiconductors such as In_1 − *x*_Mn_*x*_As, the optically excited carriers globally align the transition metal spins to induce ferromagnetism. Photoemission spectroscopy is one of the powerful techniques to observe the electronic structural changes induced by the optical excitation as demonstrated in various transition metal compounds such as spinel-type CuIr_2_S_4_[3] and perovskite-type Cs_2_Au_2_Br_6_[4]. Here, we discuss photoinduced electronic states at the surfaces of Pr_0.55_(Ca_1 − *y*_Sr_*y*_)_0.45_MnO_3_ (*y* = 0.25), SrTiO_3_, and Ti_1 − *x*_Co_*x*_O_2_ (*x* = 0.05, 0.10) on the basis of polaronic pictures.

## Methods

The ultraviolet photoemission spectroscopy (UPS) measurements were performed using a SES-100 analyzer (VG Scienta, Tokyo, Japan) with the He I line (hν = 21.2 eV) as a vacuum ultraviolet source. The energy resolution was set to 30 meV. The X-ray photoemission spectroscopy (XPS) measurements were performed using a JPS-9200 analyzer (JEOL Ltd., Akishima, Tokyo, Japan) with monochromatized AlKα (hν = 1486.6 eV) as an X-ray source. The energy resolution was set to 600 meV. Base pressures of the photoemission chambers were in the 1 × 10^−7^ Pa range. For the optical excitation, a Nd:YAG laser was used which released 10 to 30 pulse shots per second. The second and third harmonic waves (wavelengths of 532 and 355 nm) were produced and introduced into the photoemission chamber through a quartz window. The laser output per pulse was set to 0.1 to 2.0 mJ/pulse. In order to obtain a clean surface of SrTiO_3_ which is suitable for the observation of the photoinduced effect, single crystals of SrTiO_3_ should be annealed at 870 K for 20 min under oxygen pressure ranging from 3 × 10^−4^ to 7 × 10^−4^ Pa. If the crystal was annealed at 870 K with *P*_O2_ lower than 3 × 10^−4^ Pa, the crystal surface was reduced too much, and the giant photoconductivity at 20 K was lost. The samples were sandwiched by the copper plates which were grounded. The entire sample surface and the edge of the copper plate were irradiated by the laser light to establish the conducting path. High-quality thin-film samples of perovskite-type Pr_0.55_(Ca_1 − *y*_Sr_*y*_)_0.45_MnO_3_ and anatase-type Ti_1 − *x*_Co_*x*_O_2 − *δ*_ have been provided by the Miyano group, University of Tokyo and Kawasaki group, Univesity of Tokyo. The details of the Pr_0.55_(Ca_1 − *y*_Sr_*y*_)_0.45_MnO_3_ thin film are reported in [5], and those of the Ti_1 − *x*_Co_*x*_O_2 − δ_ thin film is reported in [6]. We measured the anatase-type Ti_1 − *x*_Co_*x*_O_2 − *δ*_ thin films (thickness of 40 nm) which typically show electron mobility with two orders of magnitude higher than the rutile films. Samples of *n* = 1.1 × 10^19^ and 4.5 × 10^18^ cm^−3^, and *n* = 8.7 × 10^19^ and 3.5 × 10^19^ cm^−3^ were prepared for *x* = 0.05 and 0.10, respectively, where *n* is the carrier concentration and is controlled by the amount of the oxygen deficiency *δ*. Ferromagnetism was clearly observed above room temperature in all samples except the *x* = 0.05 and *n* = 4.5 × 10^18^ cm^−3^ sample.

## Results and discussion

Pr_0.55_(Ca_1 − *y*_Sr_y_)_0.45_MnO_3_ and SrTiO_3_ have the perovskite structure. In Pr_0.55_(Ca_1 − *y*_Sr_*y*_)_0.45_MnO_3_, Mn^4+^(*d*^3^, *S* = 3/2) and Mn^3+^(*d*^4^, *S* = 2) configurations are mixed, and spin-charge-orbital orderings are expected. On the other hand, SrTiO_3_ with Ti^4+^(*d*^0^) is basically a wide-gap semiconductor. Pr_0.55_(Ca_1 − *y*_Sr_*y*_)_0.45_MnO_3_ exhibits competition between the ferromagnetic metallic (FM) state and the antiferromagnetic charge-orbital ordered insulating (COOI) state [5]. The boundary between the FM state and the COOI state is located at *y* = 0.25 in the electronic phase diagram of Pr_0.55_(Ca_1 − *y*_Sr_*y*_)_0.45_MnO_3_. At the boundary with the phase competition, Takubo et al*.* have discovered a photoinduced transition from the COOI state to the FM state using resistivity measurement under laser illumination [5]. In this context, the perovskite-type Mn oxide is one of the most important systems to study their electronic structural changes by the photoexcitation. On the other hand, SrTiO_3_ exhibits quantum paraelectricity and giant photoconductivity at low temperatures [7-10]. In the quantum paraelectric state, the dielectric constant of SrTiO_3_ dramatically increases with decreasing temperature although SrTiO_3_ does not reach ferroelectric transition even at the lowest temperature. The quantum paraelectric state is very fragile against external perturbations such as uniaxial stress, the electric field, and the isotope substitution [7-10]. As for the giant photoconductivity, the ultraviolet light irradiation induces Ti 3*d* carriers with high mobility and provides a dramatic increase of carrier density at low temperatures [11,12]. In addition, green luminescence with a long lifetime up to several milliseconds is observed under the ultraviolet irradiation at low temperatures. In an infrared absorption study by Okamura et al., a broad absorption band appears under ultraviolet light irradiation, indicating that the electronic structure of SrTiO_3_ is strongly modified by the ultraviolet light irradiation [13].

**Figure 1:**
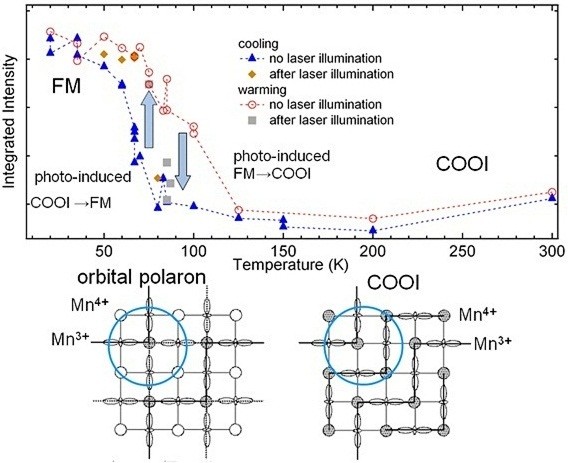
**Intensity of well-screened structure of Mn 2*p *spectra, free energy profiles, and charge and orbital arrangements.** Upper panel: integrated intensity of the well-screened structure of Mn 2*p* core-level spectra of Pr_0.55_(Ca_1 − *y*_Sr_y_)_0.45_MnO_3_ (*y* = 0.25) as a function of temperature. The integrated intensity represents the volume fraction of the FM state, and those in the cooling and warming processes are indicated by the close triangles and open circles, respectively. The integrated intensities after laser illumination are indicated by closed diamonds and closed squares. The arrows denote the changes caused by the laser illumination. Lower panel: conceptual drawings for the charge and orbital arrangements for the FM state with the ferromagnetic orbital polarons and for the COOI state.

The transition between the FM and COOI states in Pr_0.55_(Ca_1 − *y*_Sr_*y*_)_0.45_MnO_3_ can be observed in the Mn 2*p* XPS spectra as well as the valence-band photoemission spectra [14]. In particular, the intensity of the well-screened structure of Mn 2*p* spectra represents the volume fraction of the FM state, which is plotted as a function of temperature in Figure 1. The volume fraction of the FM state shows the hysteresis across the transition between FM and COOI states. In the COOI phase on the cooling process, the Mn 2*p* spectra taken after laser illumination (wavelength 532 nm) show the enhancement of the FM state, indicating that the COOI state with Jahn-Teller distortion can be changed into the FM state. On the other hand, the FM state on the warming process can be changed into the COOI state by the laser illumination above 75 K [15]. In the COOI phase of Pr_0.55_(Ca_1 − *y*_Sr_*y*_)_0.45_MnO_3_, the charge and orbital ordering is stabilized by the Jahn-Teller-type lattice distortion as shown in the lower right panel of Figure 1. When the charge and orbital ordering in the COOI state is locally destroyed by the optical excitation, the Jahn-Teller distortion in the surrounding region is weakened, and a kind of orbital polaron with ferromagnetic configuration can be formed due to the flip of the Mn 3*d e*_g_ orbitals of the Mn^3+^ sites (see the lower panel of Figure 1). The ferromagnetic orbital polaron can be expanded by the strong electron-lattice coupling in Pr_0.55_(Ca_1 − y_Sr_y_)_0.45_MnO_3_ and can evolve into an FM domain. On the other hand, the Mn 3*d* spins in the FM state can be locally flipped by the optical excitation, and consequently, the local antiferromagnetic polaron can evolve into the COOI domain. We speculate that such orbital polaron formation plays important roles in the photoinduced transitions.

**Figure 2:**
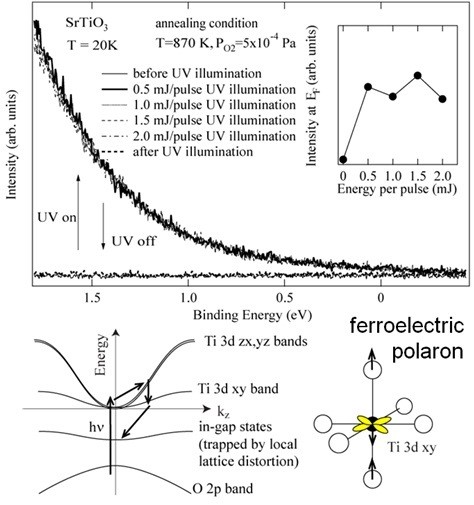
**UPS spectra and ferroelectric polaron model.** UPS spectra near the Fermi level of SrTiO_3_ are shown with conceptual diagrams for the trapping mechanism of the photoinduced carriers and for the ferroelectric lattice distortion. The UPS spectra were taken at 20 K with and without the ultraviolet illumination. The laser power is ranging from 0.5 to 2.0 mJ/pulse, and the repetition rate is 30 Hz. The inset shows the UPS spectral weight at the Fermi level as a function of the illumination power.

In order to study the unusual electronic states induced by the ultraviolet irradiation (wavelength 355 nm), we have observed the valence-band photoemission spectra of the single crystals of SrTiO_3._ Under the ultraviolet illumination, photoemission spectra were obtained without charging effect due to the giant photoconductivity. As shown in Figure 2, photoemission intensity in the bandgap region is dramatically enhanced under the ultraviolet illumination, showing that the electronic states are induced within the bandgap under the ultraviolet illumination [16]. The amount of the photoinduced electronic states within the bandgap (photoinduced in-gap states) does not change appreciably with increasing laser power from 0.5 to 2.0 mJ/pulse. Therefore, the density of the in-gap states has its maximum, and the density of electrons excited by a single shot of 0.5 mJ/pulse is enough to reach the maximum. In the present experiment, the repetition rate of 30 Hz of the ultraviolet laser is enough due to the long lifetime of the photoexcited carriers.

The photoinduced spectral weight in the bandgap is consistent with the absorption band under ultraviolet light irradiation [13]. The spectral weight in the bandgap indicates that most of the photoexcited carriers are trapped in the in-gap states. However, the tail of the in-gap states reaches the Fermi level and is probably responsible for the giant photoconductivity. As shown in the inset of Figure 2, the spectral weight at the Fermi level is increased under the ultraviolet illumination. Therefore, one can speculate that the photoinduced free carriers at the Fermi level and the electrons trapped within the bandgap coexist in SrTiO_3_ under the ultraviolet illumination. As for the electrons trapped in the bandgap, the electrons with higher binding energies are expected to be more localized due to the local lattice distortion of the TiO_6_ octahedron. This is because the more localized electrons tend to be more stabilized due to the strong electron-lattice interaction. Also, the more localized electrons with higher binding energies are expected to couple with the photocreated O 2*p* holes more strongly due to the Coulomb attractive force when the localized electrons are close to the localized O 2*p* holes.

The Ti 3*d yz*, *zx*, and *xy* electrons are stabilized due to the lattice distortions which induce the dipole moments along the *x*-, *y*-, and *z*-axes and due to the excitonic coupling with the O 2*p x*, *y*, and *z* holes. For example, under the distortion with the dipole moments along the *z*-axis, the Ti 3*d xy* electrons and the O 2*p z* holes are stabilized. The photoinduced in-gap states made up from the Ti 3*d xy* orbital are expected to induce such lattice distortion and dipole moment. Therefore, the enhancement of the dielectric constant under the ultraviolet illumination in SrTiO_3_ is probably related to the formation of the in-gap states with Ti 3*d xy* orbital polarization. This situation, which is schematically shown in Figure 2, can be viewed as a kind of ferroelectric polaron where the photoinduced Ti 3*d* electron is surrounded by the dipole moments of the distorted TiO_6_ octahedra. The ferroelectric polaron is consistent with most of the experimental results on the quantum paraelectric phase of SrTiO_3._

Ti_1 − *x*_Co_*x*_O_2 − *δ*_ has been attracting great interest since the discovery of the carrier-induced ferromagnetism at room temperature [17]. The carrier-induced ferromagnetism has been supported by various experiments including the anomalous Hall resistivity, magnetic circular dichroism, and photoemission spectroscopy [18-21]. In this mechanism, the ferromagnetism is driven by the exchange interaction between the Co spins and the n-type carriers in the Ti 3*d* band. Since Ti_1 − *x*_Co_*x*_O_2 − *δ*_ shows carrier-induced ferromagnetism, it is expected that photoinduced carriers by ultraviolet illumination cause a magnetic transition similar to In_1 − *x*_Mn_*x*_As. In this context, the photoemission study of Ti_1 − *x*_Co_*x*_O_2 − δ_ under ultraviolet illumination is very interesting and useful. In the previous photoemission study on rutile-type Ti_1 − *x*_Co_*x*_O_2 − *δ*_ thin films, the core-level peak position under ultraviolet illumination was shifted to a lower binding energy with increasing Co content *x*[19]. The energy shift was attributed to the chemical potential shift due to the increase of exchange splitting of the conduction band with increasing Co content. Here, we discuss anatase-type Ti_1 − *x*_Co_*x*_O_2 − *δ*_ thin films for various carrier concentrations *n* and Co contents *x* and their electronic structural change induced by the ultraviolet illumination.

**Figure 3:**
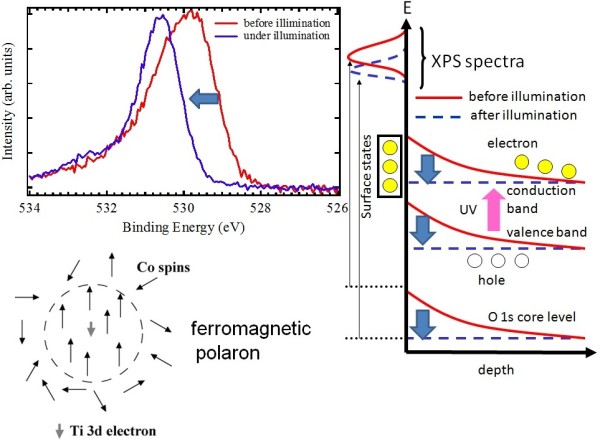
**O 1*s *core-level spectra, band bending model, and ferromagnetic polaron model.** The O 1*s* core-level spectra for *x* = 0.10 are shown with a conceptual diagram for the photoinduced change of the band bending and for the ferromagnetic polaron. The O 1*s* photoemission spectra were taken before and under the ultraviolet illumination. The arrow indicates the spectral shift and narrowing due to the illumination. The profiles of the O 1*s* core level, valence-band maximum, and conduction-band minimum are shown in the conceptual diagram.

The valence-band and core-level photoemission spectra under ultraviolet laser illumination (wavelength 355nm) show a systematic energy shift, indicating that photoinduced carriers are injected into the surface depletion layer to enhance their ferromagnetism [22]. Figure 3 shows the O 1*s* core-level spectra for the *x* = 0.10 sample taken before and under the ultraviolet laser illumination. Under the ultraviolet illumination, the O 1*s* core-level peak position is shifted to the high binding energy side. Since the illumination of the visible light (wavelength of 532 nm) does not induce any change to the O 1*s* core-level spectra, the optical excitation across the bandgap of TiO_2_ plays important roles in the core-level energy shift and the spectral sharpening. In addition to this energy shift, the spectrum is sharpened under the ultraviolet illumination. The energy shift and the spectral sharpening by the ultraviolet illumination depend on the carrier concentration *n* and the Co content *x*. When *x* is fixed and the carrier concentration *n* is varied, the difference in *n* affects the spectral sharpening but does not affect the magnitude of the energy shift. The optical excitation across the bandgap due to the ultraviolet illumination provides photoinduced carriers and can induce surface photovoltage or reduction of band bending near the surface. The spectra under the ultraviolet illumination did not show any additional changes by further ultraviolet illumination higher than 1.0 mJ/pulse. This indicates that the band bending is almost eliminated with the ultraviolet illumination of 1.0 mJ/pulse. The right panel of Figure 3 schematically shows the elimination of band bending. Before the ultraviolet illumination, the excess charge associated with surface states is compensated by the charge within a depletion layer, leading to the band bending. Under the ultraviolet illumination, the electron–hole pairs created by the optical excitation are separated by the electric field of the depletion region to induce the surface photovoltage. The magnitude of the O 1*s* energy shift corresponds to the magnitude of the band bending determined by the charge density of the surface states before the ultraviolet illumination. The magnitude of band bending is given by the electrostatic potential *φ*(*z*) = −*ne*(*z* − *d*)^2^/2*ε*, where the depth of depletion layer *d* = (2*εφ*_0_/*ne*)^1/2^, the *z*-axis is perpendicular to the surface, *ε* is the dielectric constant, and *φ*_0_ is the magnitude of the electrostatic potential at the surface (*z* = 0). The energy shift induced by the ultraviolet illumination corresponds to *φ*_0_, assuming that the band bending is completely eliminated under the ultraviolet illumination. The values of *d* calculated from the equation are 7.5, 11.8, 18.2, and 28.5 nm for *n* = 8.7 × 10^19^, 3.5 × 10^19^, 1.1 × 10^19^, and 4.5 × 10^18^ cm^−3^. It is expected that the magnitude of *n* is related to the spectral broadening before the ultraviolet illumination through *dφ*(*z*)/*dz* at *z* = 0 that is given by (2*neφ*_0_/*ε*)^1/2^*.* Indeed, the photoemission spectral width correlates with *n*, supporting this interpretation.

Interestingly, the shifted and sharpened O 1*s* spectra are persistent even after switching off the ultraviolet illumination. Before the ultraviolet illumination, there are no carriers in the depletion layer due to the band bending, and the Co spins in the depletion layer are disordered. Under the ultraviolet illumination, the band bending is removed by the photoinduced Ti 3*d* carriers, and the Co spins in the depletion layer are aligned by the carriers which are injected into the depletion region. The Ti 3*d* carriers are expected to form ferromagnetic polarons as illustrated in Figure 3. Such ferromagnetic polarons are locally very stable, and consequently, the shifted and sharpened O 1*s* spectra are persistent even after switching off the ultraviolet illumination. Specifically, due to the strong interaction between the carriers and the Co spins, the ferromagnetic state and the insulating paramagnetic state almost degenerate in energy, and the small amount of photoinduced carriers can cause the drastic change from the insulating and paramagnetic surface with the depletion layer to the metallic and ferromagnetic surface without the depletion layer.

## Conclusions

The unusual electronic states induced by laser illumination in SrTiO_3_, Pr_0.55_(Ca_1 − *y*_Sr_*y*_)_0.45_MnO_3_ (*y* = 0.25) and Ti_1 − *x*_Co_*x*_O_2 − *δ*_ (*x* = 0.05, 0.10) have been discussed on the basis of various polaronic pictures. In Pr_0.55_(Ca_1 − *y*_Sr_y_)_0.45_MnO_3_ (*y* = 0.25), the visible light irradiation can cause the metal-to-insulator and insulator-to-metal transitions in which Mn 3*d* orbital polarons play important roles. SrTiO_3_ shows the in-gap state under the ultraviolet illumination which is assigned to the localized electronic state with polarized lattice distortion. This state can be interpreted as a ferroelectric polaron state. In Ti_1 − *x*_Co_*x*_O_2 − δ_ thin films, the band bending at the surface region is removed by the ultraviolet illumination, indicating the possibility of the photoinduced ferromagnetism in the surface region at room temperature. The persistent change can be explained by the stability of the ferromagnetic polaron in the diluted magnetic semiconductor. In the future, various photoinduced phase transitions at surfaces or interfaces of transition metal oxides should be explored more systematically using spectroscopic and theoretical approaches.

## Competing interests

The author declares that he has no competing interests.

## Author’s information

TM is an associate professor at Graduate School of Frontier Sciences, University of Tokyo.
